# Insights into idarubicin antimicrobial activity against methicillin-resistant *Staphylococcus aureus*

**DOI:** 10.1080/21505594.2020.1770493

**Published:** 2020-05-29

**Authors:** Pengfei She, Shijia Li, Linying Zhou, Zhen Luo, Jinfeng Liao, Lanlan Xu, Xianghai Zeng, Ti Chen, Yaqian Liu, Yong Wu

**Affiliations:** Department of Clinical Laboratory, The Third Xiangya Hospital of Central South University, Changsha, R.P. China

**Keywords:** Drug repurposing, idarubicin, skin and soft tissue infections, methicillin-resistant *Staphylococcus aureus*, topoisomerase II, cell membrane

## Abstract

**Background:**

MRSA is a major concern in community settings and in health care. The emergence of biofilms and persister cells substantially increases its antimicrobial resistance. It is very urgent to develop new antimicrobials to solve this problem.

**Objective:**

Idarubicin was profiled to assess its antimicrobial effects *in vitro* and *in vivo*, and the underlying mechanisms.

**Methods:**

We investigated the antimicrobial effects of idarubicin against MRSA by time-kill analysis. The antibiofilm efficacy of idarubicin was assessed by crystal violet and XTT staining, followed by laser confocal microscopy observation. The mechanisms underlying the antimicrobial effects were studied by transmission electron microscopy, all-atom molecular dynamic simulations, SYTOX staining, surface plasma resonance, and DNA gyrase inhibition assay. Further, we addressed the antimicrobial efficacy in wound and subcutaneous abscess infection *in vivo*.

**Results:**

Idarubicin kills MRSA cells by disrupting the lipid bilayers and interrupting the DNA topoisomerase IIA subunits, and idarubicin shows synergistic antimicrobial effects with fosfomycin. Through synergy with a single dose treatment fosfomycin and the addition of the cell protector amifostine, the cytotoxicity and cardiotoxicity of idarubicin were significantly reduced without affecting its antimicrobial effects. Idarubicin alone or in combination with fosfomycin exhibited considerable efficacy in a subcutaneous abscess mouse model of MRSA infection. In addition, idarubicin also showed a low probability of causing resistance and good postantibiotic effects.

**Conclusions:**

Idarubicin and its analogs have the potential to become a new class of antimicrobials for the treatment of MRSA-related infections.

## Introduction

*Staphylococcus aureus* is one of the most common gram-positive opportunistic human pathogens, which is carried by approximately 30% of the human population [1]. Despite antibiotic availability, *S. aureus*-related infections often remain one of the main causes of death and are difficult to cure [2]. The relatively high burden of methicillin-resistant *S. aureus* (MRSA) in community and health-care settings is a major concern worldwide [3]. Additionally, the emergence of linezolid (LZD) [4] and vancomycin (VAN) [3] resistant strains has been sporadically reported worldwide. In addition, the emergence of persister cells and biofilms significantly increases antimicrobial resistance, which makes conventional antibiotics that target cellular growth processes ineffective, resulting in high clinical failure rates of antibiotic therapy [5].

*S. aureus* causes many kinds of human infections and syndromes, with the most common forms of infection being skin and soft tissue infections (SSTIs) [6]. Among them, the most common bacterial infections in children are acute bacterial skin and skin structure infections [7]. In military personnel with these types of infections, 91% are caused by *S. aureus* (70% of them are MRSA strains), with 4–6% ultimately experiencing an SSTI [8]. Moreover, high bacterial loads of greater than 10^8^ CFU/ml are present in SSTIs, highlighting the importance of investigating high bacterial density infections. SSTIs, such as pus-filled pockets infiltrated by immune cells and bacteria, also known as abscesses, form fluid and are often highly resistant to conventional antibiotic treatment. Thus, the development of new antibiotics against persistent *S. aureus* is an urgent issue [9].

The development of new antimicrobials is a very slow process and is frequently beset with a large number of obstacles [10,11]. Repurposing approved drugs is a promising alternative strategy, and these drugs have the ability to go directly to preclinical and clinical trials, reducing cost and time [12].

Idarubicin (IDAR) is a type of anthracycline antibiotic used for acute myeloid leukemia therapy [13]. Mammalian DNA topoisomerase II is the target of anticancer anthracyclines that act by stabilizing enzyme-DNA complexes wherein DNA strands are cut and covalently linked to the protein. This molecular mechanism is the basis of anthracycline anticancer activity, as well as the toxic effects [14]. It has been demonstrated that IDAR and doxorubicin can inhibit the growth of gram-positive cocci in blood culture bottles *in vitro* [15,16]. However, there has been no systematic study on the antimicrobial mechanisms of IDAR or its *in vivo* efficacy.

Thus, in the present study, we present a detailed bioanalysis of IDAR as an antimicrobial, including its mechanism and *in vivo* efficacy against MRSA in acute skin infection and subcutaneous abscess infection models. Then, we attempted to decrease the cytotoxicity of IDAR by topically use with the addition of the cell protector amifostine (AMI) and by combination with fosfomycin (FOS).

## Materials and methods

### Bacterial strains and growth conditions

The details of bacterial strains and growth conditions were described at Supplementary methods 1.

### Antimicrobial susceptibility tests using the microdilution assay

The minimal inhibitory concentration (MIC) and minimal bactericidal concentration (MBC) of antibiotics and IDAR were determined using the microdilution assay strictly according to the recommendations of the Clinical & Laboratory Standards Institute (CLSI) [17].

### Disc diffusion assay

An overnight culture of *S. aureus* was diluted in fresh TSB broth and cultured to mid-log phase. The suspension was diluted to the equivalent of 0.5 McFarland standard and then 150 μl was spread onto an MH agar plate. After air drying for 10 min, disks loaded with 5 μg ciprofloxacin (CIP), 20 μg VAN, or 20 μg IDAR were placed onto the agar. Disks loaded with 2% DMSO were included as a negative control. The plates were then incubated at 37°C for 16–18 h. The diameter of the inhibitory circle was measured with a caliper [18].

### Killing kinetics assay

Tubes containing MH broth were inoculated with a suspension of *S. aureus* to a final count of approximately 2 × 10^6^ CFU/mL. The tubes were then incubated at 37°C, 200 rpm. Viable counts and culture turbidity were determined by plate counts and by measuring the optical density at 630 nm (OD630), respectively, at time points 0, 2, 4, 8, 12, and 24 h after the addition of IDAR at the indicated concentrations [19].

### Persister killing assay

To obtain persisters from a biofilm culture, a mid-log growth-phase culture of *S. aureus* was diluted in BHI broth to 1 × 10^8^ CFU/ml. One hundred microliters of this suspension was transferred to the wells of a microplate. The plates were then sealed with parafilm and incubated at 37°C in a humidified atmosphere for 24 h. Planktonic bacteria were removed by washing twice with 1 × PBS (pH = 7.0), and biofilms were exposed to 100 μl of BHI broth containing rifampicin (RFP, 100 × MIC). After 24 h of incubation at 37°C, planktonic cells were removed and any remaining attached bacteria were dislodged in 100 μl of PBS by sonication. Bacteria were subsequently exposed to 200 μl of PBS containing a 2 × MIC of IDAR. Bacteria exposed to PBS without IDAR were included as a control. The number of viable cells was determined by plate counts every 2 h for a total of 6 h [20].

### Biofilm inhibition assay

Overnight cultures of MRSA were diluted 1:200 in fresh TSB broth with or without antimicrobials at intended concentrations ranging from 0.125 to 128 μg/ml, and then 200 μl were inoculated into the wells of microplates in triplicate. After static incubation at 37°C for 24 h, the planktonic cells were removed by washing with PBS, and the remaining biofilms were stained with 0.5% crystal violet (CV) for 5 min, followed by removal by washing with saline. The CV stain was solubilized with 95% ethanol for 15 min, and the biofilm biomass was determined by measuring absorbance at 570 nm (A570) [21].

### Biofilm eradication assay

Biofilms formed as described above were treated with 200 μl of antimicrobials at intended concentrations. After incubation at 37°C for another 24 h, the remaining biofilms were measured by CV staining as described above, or by XTT assays. For the XTT [(2,3-Bis-(2-methoxy-4-nitro-5-sulfophenyl)-2 H-tetrazolium-5-carboxanilide)] assay [22], 200 μl of a solution containing 200 μg/ml of XTT and 20 μg/ml of phenazine methosulfate (PMS) was added to each well. After incubation for 3 h at 30°C in the dark, the absorbance was measured at 490 nm (A490).

### All-atom molecular dynamics (MD) simulations

The structure of the *S. aureus* cell membrane was constructed by CHARMM-GUI software. We used the previously established model of 1,2-dioleoyl-sn-glycero-3-phosphocholine (DOPC)/1,2-dioleoyl-sn-glycero-3-phospho-(1ʹ-rac-glycerol) (DOPG) at a 7:3 ratio [23]. After filling water molecules and countering ions, equilibrium simulation was performed by Gromacs 2018.4 [24] within 200 ns. The structure information was downloaded from NCBI and the RESP electric charges were calculated at the level of B3LYP/def2TZVP [25]. MD was performed with Gromacs 2018.4 software, hydrogen bonds were restrained by the LINCS algorithm [26], and the interaction of the Particle-mesh Ewald was calculated by the PME algorithm [27]. The cutoff value for nonbonding interactions was set as 10 Å and was refreshed every 10 steps. The simulation temperature and pressure were control by the V-rescale [28] and Parrinello-Rahman [29] methods, respectively. MD was performed within 500 ns, and the conformation was stored per 20 ps. The simulation results were visualized by the Gromacs and VMD program.

### Molecular docking assay

Molecular docking was conducted as reported by Werner et al. [30] with minor modifications. The details of molecular docking were described at Supplementary methods 2.

### Protein synthesis and surface plasma resonance assay

The *gyrA* and *gyrB* genes were amplified by PCR and ligated into the Ndel/Xhol cloning sites of the pET-22b(+) and pET-28a(+) plasmids, respectively. The His*6 and His*10-sumo tags were added to the C-terminal end of *gyrA* and the N-terminal end of *gyrB*, respectively, and the gyrB tag was cleaved by TEV. The plasmid was then transferred to competent *E. coli* (BL21). After incubation at 37°C for 14 h, a single colony was picked and cultured to log phase, after which IPTG was used to induce the expression of *gyrA* and *gyrB*. After incubation at 18°C for another 16 h, the bacterial cells were lysed by ultrasonification in 20–30 ml Tris-HCl (10 mM, pH 8.0). After centrifugation, the proteins were collected and purified using a Ni affinity chromatography column. The surface plasma resonance (SPR) was performed with Biacore S200 and bio-sensor chip S-CM5 (Supplementary methods 3).

## Enzyme inhibition assay

Supercoiled and relaxed DNA of pBR322 were purchased from Fenghui Biotechnologies Inc. (Changsha, China). *S. aureus* DNA gyrase and 5× reaction buffer [40 mM Hepes KOH (pH 7.6), 500 mM potassium glutamate, 2 mM ATP, 0.05 mg/ml albumin, and 10 mM DTT] were obtained from Enzo Biochem (New York, America). The efficacy of DNA gyrase to supercoil relaxed DNA in the presence of ciprofloxacin (CIP, positive control) and IDAR was determined by gel electrophoresis [30,31].

### Bacterial membrane permeability assay

Log-phase bacterial suspensions in MH broth were centrifuged for 10 min at 3000 × g and resuspended in 2 mL HEPES buffer (10 mM, pH = 7.4, 150 mM NaCl) to a final concentration of approximately 5 × 10^6^ CFU/ml. Then, twofold dilutions of IDAR from 0.5 to 32 μg/mL were added to the suspensions. After incubation at 37°C at 200 rpm for 1 h, SYTOX Green was added to a final concentration of 0.1 μM and the samples were incubated for 10 min on the ice, protected from light. The intensity of fluorescence was detected by flow cytometry (BD FACSCanto) (San Jose, CA, USA). A viability assay by plate counting was performed to eliminate the influence of cell lysis on permeability [32].

### Drug combination assay

The synergistic interaction between IDAR and antibiotics of different classes was determined using the checkerboard method. Diluted bacterial cells at mid-log phase (1 × 10^6^ CFU/ml) were dispensed into microtiter plates and a two-dimensional checkerboard with twofold dilutions of IDAR and other antibiotics (FOS, amikacin (AMK), CIP, teicoplanin (TEC), gentamycin (GEN), VAN) were set up. The results were determined after 16–20 h of incubation at 37°C by measuring the OD630. The interaction was assessed by determining the optimal fractional inhibitory concentration index (FICI) using the following equation: FICI = FIC_A_+FIC_B_ (FIC = MIC in combination/MIC alone). The FICI was interpreted as follows: FICI<0.5, indicates synergism; 0.5≤ FICI<1, indicates partial synergy; FICI = 1, indicates additive effects; FICI>4, indicates antagonism [33].

### Confocal laser scanning microscopy (CLSM)

Biofilms formed on coverslips were rinsed with PBS to remove unattached cells, then stained with a mix of SYTO9 (for all cells) and PI (for dead cells) (Thermo Fisher Scientific, Shanghai, China). The coverslips were imaged using a CLSM (Zeiss LSM 800, Jena, Germany), and biofilm quantification was performed with ZEN 2012 software [34].

### Transmission electron microscopy (TEM)

*S. aureus* ATCC 43300 was grown to exponential phase at 37°C, 200 rpm. The bacterial cells were treated with IDAR at a concentration of 20 μg/ml (5 × MIC) at 37°C, 200 rpm for 1 h. After cell collection by centrifugation, the TEM was performed (Supplementary methods 4).

### Resistance induction assay

The details of resistance induction assay were described at the Supplementary methods 5.

### Animals

The project and animal experiment were approved by the Ethics Committee of the Third Xiangya Hospital of Central South University (No. 2019sydw0211). The details of animal experiments were described at the Supplementary methods 6.

## Results

### Bactericidal activity of IDAR against MRSA and *S. aureus* persisters

IDAR showed strong bactericidal effects against *S. aureus* (including MSSA and MRSA), and *S. epidermidis* with MICs and MBCs of 2–4 μg/ml and 4–32 μg/ml, respectively. Though IDAR alone only showed weak or no antimicrobial effects against gram-negative bacteria such as *A. baumannii* with an MIC of 32 μg/ml, and *E. coli* and *K. pneumoniae* with MICs >128 μg/ml. However, when combined with sub-MIC levels of polymyxin B nonapeptide (PMBN), an analog of polymyxin B with strong cell permeability ability but weak antimicrobial effects [35], the MICs of *A. baumannii, E. coli*, and *K. pneumoniae* were decreased to 16, 32, and 128 μg/ml, respectively. Additionally, *C. albicans* showed moderate susceptibility with an MIC and MBC of 64 μg/ml. However, there were no effects on *E. faecalis* or *P. aeruginosa* in the presence/absence of PMBN with MICs >128 μg/ml (Table 1). Similarly, IDAR also exhibited strong bactericidal activity against the MSSA, MRSA, and methicillin-resistant *S. epidermidis* (MRSE) clinical isolates with MICs of 1–4 μg/ml (Table S1).

**Table 1.** Antimicrobial susceptibility test.

Strains	MIC (μg/ml)	MBC (μg/ml)
***S. aureus***		
**MSSA**		
ATCC25923	4	8
Newman	2	4
**MRSA**		
ATCC43300^a^	4	8
MIC50 (*n* = 26)	4	
MIC90 (*n* = 26)	2 ~ 4	
***S. epidermidis***		
RP62A^a^	4	32
ATCC12228	4	8
***E. faecalis***		
ATCC29212^a^	>128	>128
***A. baumannii***		
ATCC1195	32	128
+PMBN	16	128
***E. coli***		
ATCC25922	>128	>128
+PMBN	32	64
***P. aeruginosa***		
PAO1^a^	>128	>128
+PMBN	>128	>128
***K. pneumoniae***		
ATCC700603	>128	>128
+PMBN	128	128
***C. albicans***		
ATCC14053	64	64

^a^biofilm-forming strains.

MSSA: Methicillin-sensitive *Staphylococcus aureus.*

MRSA: Methicillin-resistant *Staphylococcus aureus.*

**Figure 1.**
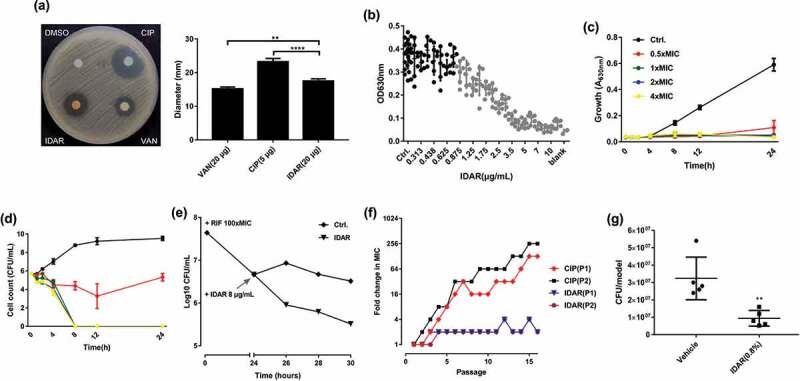
IDAR exhibits bactericidal activity against MRSA and persister cells. (a) Disk diffusion assay of IDAR showing activity against *S. aureus* ATCC 43300. The amounts of VAN, CIP, and IDAR were 20 μg, 5 μg, and 20 μg per disk, respectively. (b) Dose-dependent efficacy of IDAR against ATCC 43300 determined by the broth microdilution assay. (c) Cell growth inhibitory effects of IDAR at 0.5–4 × MIC against ATCC 43300 at different timepoints. (d) Time-dependent bactericidal effects of IDAR against ATCC 43300 at concentrations ranging from 0.5 to 4 × MIC. (e) Persister cells induced by RIF at 100 × MIC, followed by treatment with 8 μg/ml IDAR for a total of 6 h. Viable cell counts were recorded by the serial dilution method. (f) Appearance of ATCC 43300 spontaneous resistance in the presence of IDAR and CIP over 16 d of serial passage in duplicate (P1 and P2). (g) MRSA antimicrobial effects of 8 h of treatment with an ointment containing IDAR (0.8%, wt/wt) in an acute wound model infected by ATCC 43300.

Using the disc dilution method, MRSA was more susceptible to IDAR (20 μg/disc) compared to VAN (20 μg/disc), but less susceptible than CIP (5 μg/disc) (Figure 1(a)). IDAR showed dose-dependent antimicrobial effects against MRSA from 0.875 to 5 μg/ml (Figure 1(b)). IDAR could inhibit cell growth at concentrations of ≥0.5 × MIC (Figure 1(c)). Additionally, strong bactericidal effects were observed with IDAR concentrations ≥1 × MIC, with the bacterial cells completely dead within 8 h (Figure 1(d)). And 10-fold MIC of IDAR still showed stronger bacteriostatic and even bactericidal activity even in the presence of high density of MRSA cells (approximately 10^8^ CFU/ml) than 10-fold MIC of VAN (Fig. S1 A, B). We found that a 2 × MIC of IDAR could eradicate nearly 100-fold number of persister cells induced by 24 h treatment with a 100 × MIC of RFP, compared to untreated persisters after 6 h (Figure 1(e)). Sub-MIC levels of CIP could induce antibiotic resistance via the stress response, and we found that the MIC of CIP was increased by up to 256-fold in the presence of sub-MIC values of CIP over 16 passages. However, IDAR still maintained nearly the same susceptibility profile among cells in the presence of sub-MIC values of IDAR for 16 passages (Figure 1(f)). In addition, the occurrence of resistant high-density bacterial loads (approximately 10^9^ CFU/plate) was significantly inhibited by IDAR at concentrations ≥2 × MIC (Table 3). In an *in vivo* acute MRSA infection wound model, 8 h of treatment with 0.8% IDAR could reduce the bacterial load by nearly threefold compared to the vehicle group (Figure 1(g)).

**Table 2.** FICs of antimicrobials against coci.

Agent	MIC(ug/mL)		MIC_In combination_/MIC_singly_	FIC_opt_	Outcome
	Singly	In combination			
FOS	16	2	0.125	0.25	Synergy
IDAR	4	0.5	0.125		
AMK	64	16	0.25	0.375	Synergy
IDAR	4	0.5	0.125		
CIP	1	0.5	0.5	0.75	Partial synergy
IDAR	4	1	0.25		
TEC	0.5	0.25	0.5	0.75	Partial synergy
IDAR	4	1	0.25		
GEN	1024	128	0.25	0.75	Partial synergy
IDAR	4	2	0.5		
VAN	1	0.5	0.5	1	Addition
IDAR	4	2	0.5

**Table 3.** Frequency of resistance to IDAR.

Drug	baseline MIC (μg/ml)	1× MIC	2× MIC	4× MIC	8× MIC
IDAR	4	5.6 × 10^−8^	<1.0 × 10^−9^	<1.0 × 10^−9^	<1.0 × 10^−9^
RIP	0.016	1.7 × 10^−8^	4.08 × 10^−8^	1.0 × 10^−8^	4.15 × 10^−8^

### IDAR inhibits biofilm formation and eradicates preformed biofilms

**Figure 2.**
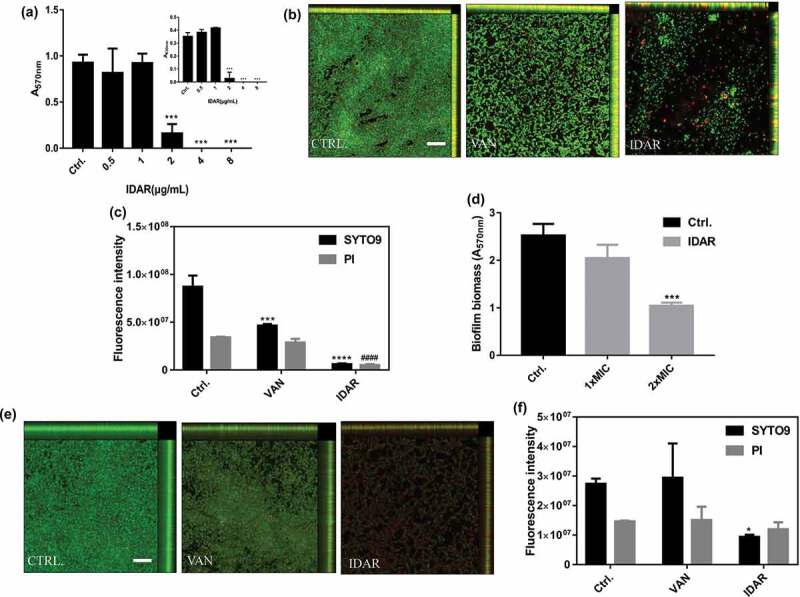
IDAR inhibits biofilm formation and eradicates preformed biofilms. (a) Dose-dependent biofilm inhibitory effects of IDAR against MRSA biofilms by the CV staining method. (b) Representative CLSM images and (c) fluorescent intensity quantification by ZEN 2012 software of IDAR biofilm inhibitory effects. The concentration of both VAN and IDAR was 2 μg/ml. (d) Biofilm eradication by IDAR as demonstrated by the CV staining method. (e) Representative CLSM images and (f) fluorescent intensity quantification by ZEN 2012 software of IDAR biofilm eradication effects.

IDAR could significantly inhibit MRSA biofilm formation at concentrations ≥2 μg/ml in a dose-dependent manner (Figure 2(a)). This result indicates that the biofilm inhibitory effects of IDAR were mainly due to its bactericidal efficacy as shown in Figure 1(b). Additionally, CLSM images showed that IDAR had stronger biofilm inhibitory effects than VAN at the concentration of 2 μg/ml (Figure 2(b)). In accordance with the CLSM images, the fluorescence intensity analysis also showed strong biofilm inhibitory effects of IDAR against MRSA (Figure 2(c)). Similarly, IDAR could also eradicate mature biofilms at a concentration of 2 × MIC (Figure 2(d)). CLSM images also showed that 8 μg/ml of IDAR had more significant biofilm eradicating effects compared to VAN at a concentration of 32 μg/ml (Figure 2(e)). Similarly, fluorescence intensity analysis showed the strong biofilm eradicating effects of IDAR (figure 2(f)).

### IDAR induces *S. aureus* cell membrane permeabilization and interferes with the topoisomerase IIA subunits GyrA and GyrB

**Figure 3.**
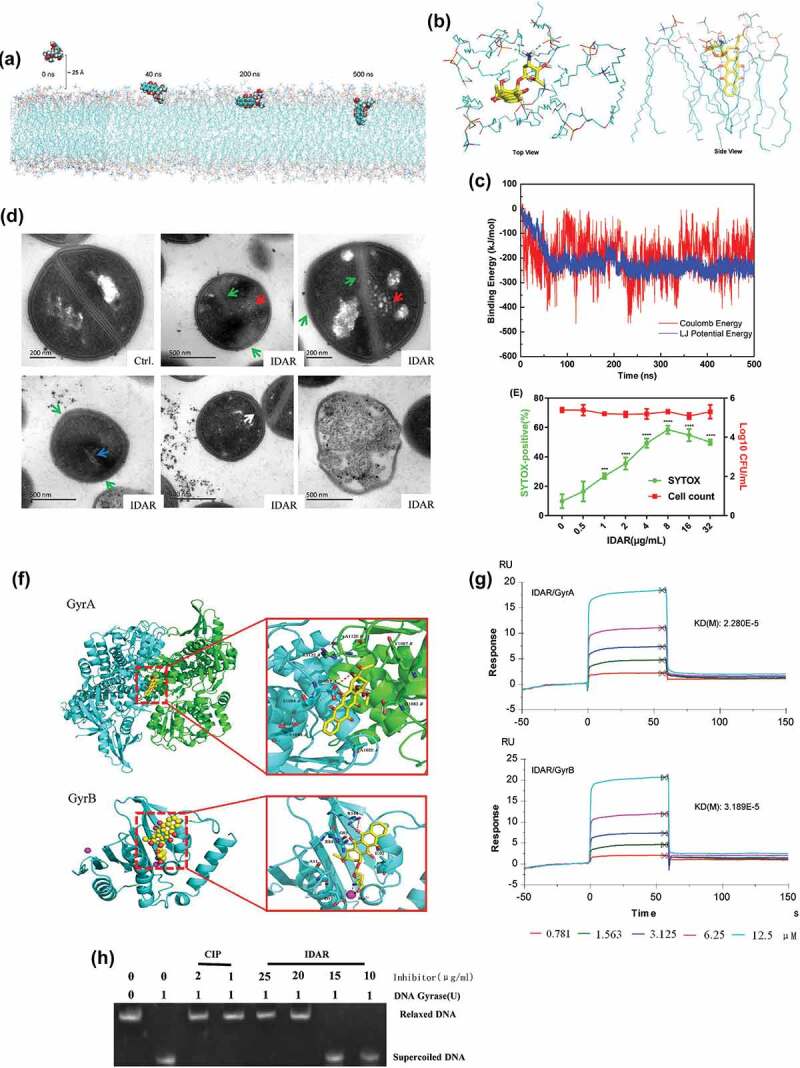
IDAR disrupted the cell membrane and interacted with the DNA Topo IIA subunits GyrA and GyrB. (a) Representative configurations of MD simulations of IDAR from left to right: onset, membrane attachment, membrane penetration, and equilibrium interaction with 7DOPC/3DOPG lipid bilayers. (b) Interaction model between IDAR and phospholipid groups. (c) The free-energy profiles of IDAR penetrating into the indicated lipid bilayers as a function of the interaction time with the bilayer. (d) TEM showing mesosome-like structures (red arrow), cell wall disruption (green arrow), abnormal intracellular aggregation (blue arrow), cell membrane disruption (white arrow), and cell lysis in 5 × MIC IDAR-treated cells and DMSO controls. (e) Uptake of SYTOX Green (green line) and viable cell counts (red line) by exponential-phase *S. aureus* ATCC 43300 cells treated with IDAR. (f) Model of the interaction between IDAR and GyrA and GyrB by molecular docking analysis. (g) Affinity between IDAR and the GyrA and GyrB proteins by SPR analysis. (h) Inhibition of *S. aureus* gyrase activity by CIP and IDAR.

Using all-atom MD simulations of IDAR interacting with bacterial membranes, we found that IDAR is initially recruited to the membrane surface and after several hundred nanoseconds (approximately 40 ns) of sustained attachment, the centroid distance between IDAR and membranes is getting closer, which shows that the small molecules can quickly attach to the surface of phospholipid membrane, and tend to be stable (Fig. S2A). IDAR then penetrates into the membrane interior. After penetration, IDAR embeds into the outer leaflet of the lipid bilayer and disrupts the integrity of the cell membrane (Figure 3(a)). The number of hydrogen bonds between IDAR and phospholipid membrane is stable at about 2. It shows that the hydrogen bond and electrostatic interaction between amino group and phosphate group can stabilize the combination (Fig. S2B, Supplementary Video at https://v.youku.com/v_show/id_XNDQ0MDM5NjYyMA==.html?spm=a2h3j.8428770.3416059.1). The 3-dimensional binding mode showed that small positively charged amino heads form stable hydrogen bonds with negatively charged phosphoric acid groups in phospholipid membranes, while aromatic tails extend into the middle of highly hydrophobic phospholipid membranes, forming strong hydrophobic interactions (Figure 3(b)). Additionally, the bacterial membrane had a low energy barrier toward IDAR and highly favorable transfer energies (Figure 3(c)). TEM images showed the formation of mesosome-like structures, abnormal cell division, abnormal intracellular aggregations, cell membrane damage, and cell lysis in the presence of 5 × MIC of IDAR (Figure 3(d)). Using an SYTOX Green permeability assay, we found that IDAR induced dose-dependent membrane permeability within 1 h without significant cell viability changes, which indicates that the bactericidal activity of IDAR could be initiated by targeting the cell membrane (Figure 3(e)). To investigate the binding mode of IDAR with proteins, docking simulation studies were carried out. We found that IDAR exhibited better docking model with GyrA and GyrB compared to its intrinsic ligand (Fig. S3-S5 and Table S2). The binding mode of IDAR with GyrA/GyrB is illustrated in Figure 3(f). We found that IDAR interacts with Asp1083 in chain A of GyrA through a hydrogen bond interaction, and with Arg144 and Mg2+ of GyrB through hydrogen bond interactions and ion contacts, respectively. The computational results indicated that IDAR can interact with GyrA and GyrB. Additionally, in an SPR study, we found that IDAR could significantly interact with GyrA and GyrB with KD(M) values of 2.280E-5 and 3.189E-5, respectively (Figure 3(g)). And the inhibitory effect of IDAR against DNA gyrase activity was also determined by DNA motility assay. Figure 3(h) shows that the CIP (positive control) showed a significant inhibitory effect against the DNA gyrase at the concentration of 1 μg/ml. However, 20 μg/ml of IDAR was needed to reach the same inhibitory effect, which indicates the antimicrobial effects of IDAR probably mainly due to its disruption with the cell membrane.

### IDAR shows synergistic antimicrobial effects with FOS against *S. aureus*

**Figure 4.**
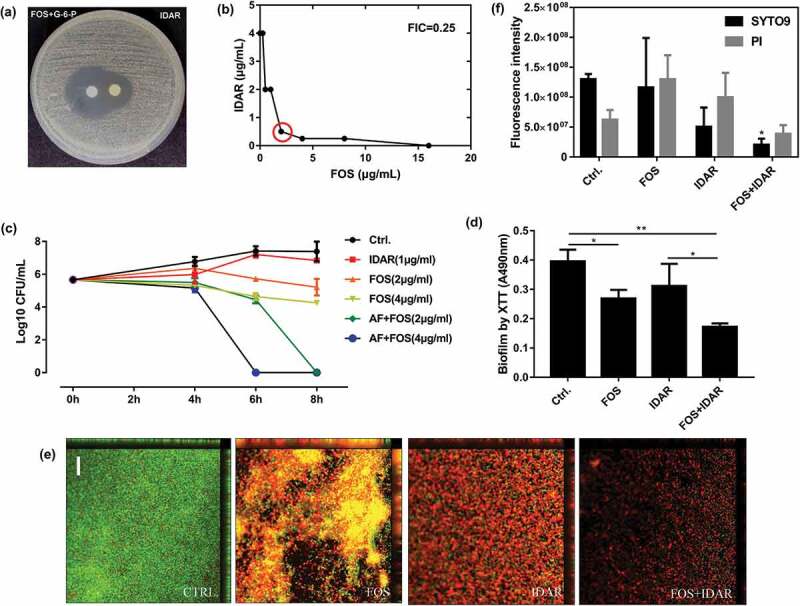
Synergistic effects between IDAR and FOS against MRSA planktonic cells and biofilms. (a) Drug interaction determined by a disk diffusion assay. (b) Drug interaction determined by a checkerboard assay. The red circle indicates the optimal combination with the lowest FICI. (c) Time-kill curve of drugs in combination or used alone. (d) Antibiofilm effects by IDAR and FOS alone or in combination demonstrated by XTT staining. (e) Representative CLSM images of antibiofilm effects of IDAR and FOS alone or in combination.

Checkerboard assays demonstrated that IDAR showed significant synergistic effects with FOS and AMK with FICIs of 0.25 (Figure 4(b)) and 0.375, respectively. Partial synergy effects were also observed with CIP, TEC, and GEN with FICIs of 0.75, an additive effect was observed with VAN with an FICI of 1 (Table 2). Due to the low MIC values after the combination of IDAR and FOS, FOS was selected for further research. The disc dilution method confirmed the synergistic effects between IDAR and FOS (Figure 4(a)). Using time-killing assay, we found that sub-MICs of IDAR or FOS showed no or only minimal bactericidal effects against MRSA; however, sub-MIC levels of IDAR could eradicate nearly 100% of live bacterial cells within 6 and 8 h when combined with 2 and 4 μg/ml FOS, respectively (Figure 4(c)). Similarly, by XTT assay, we found that FOS could also enhance the biofilm eradicating effects of IDAR (Figure 4(d)). CLSM images also demonstrated the synergistic antibiofilm effects of IDAR and FOS (Figure 4(e)). By fluorescence intensity analysis, a single dose of IDAR and FOS showed no effects on MRSA biofilms; however, in combination, the total MRSA biofilm biomass was significantly decreased (stained by SYTO9) and the proportion of dead cells was significantly increased (stained by PI) (Figure 4(e)).

### AMI relieved the cytotoxicity of IDAR

**Figure 5.**
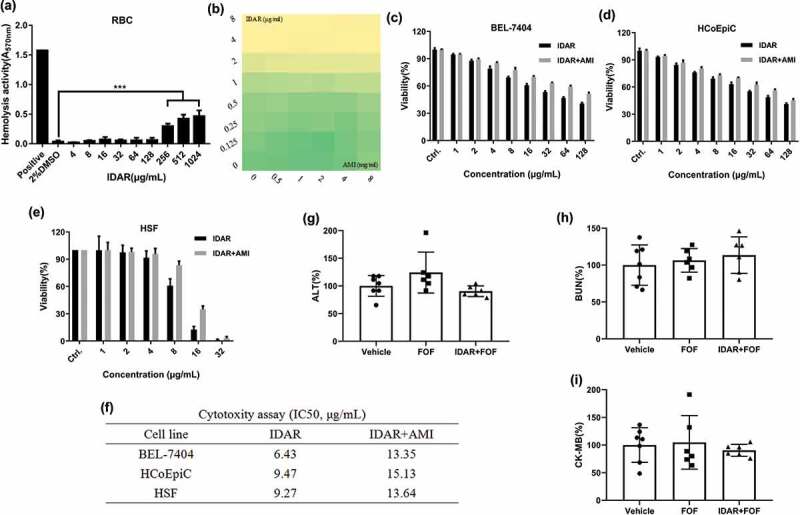
AMI relieved the cytotoxicity of IDAR in mammalian cells. (a) Sheep blood hemolytic activity of IDAR. DMSO was included as a control. (b) Effects of AMI on the antimicrobial effects of IDAR determined by a checkerboard assay. (c) BEL-7404, (d) HcoEpiC, and (e) HSF IDAR cytotoxicity determined by CCK-8 assays. (f) IC50 of IDAR in the presence/absence of AMI. *In vivo* toxicity of IDAR to the liver (g), kidney (h), and cardiac system (i) after 24 h of treatment in a mouse model.

Hemolytic tests were performed with horse RBCs, and we found that IDAR showed no hemolytic activity on RBC membranes below a concentration of 128 μg/ml indicating that there was no interaction between IDAR and the RBC cell membrane (Figure 5(a)). AMI is a kind of broad-spectrum cell protective agent, which showed no interaction with IDAR and no influence on its antimicrobial activity (Figure 5(b)). While IDAR inhibited the viability of the BEL-7404 (Figure 5(c)), HCoEpiC (Figure 5(d)) and HSF (Figure 5(e)) cell lines in a dose-dependent manner, with the 50% inhibitory concentrations (IC50) of 6.43, 9.47, and 9.27 μg/ml, respectively, the addition of 14 mM AMI significantly relieved its cytotoxicity and increased the IC50s to 13.35, 15.13, and 13.64 μg/ml, respectively (Figure 5(f)). Similarly, FOS alone or in combination with IDAR + AMI showed no *in vivo* toxicity against the liver (Figure 5(g)), kidney (Figure 5(h)), or heart (Figure 5(i)) compared to the vehicle group.

### IDAR shows efficacy in a subcutaneous abscess mouse model

**Figure 6.**
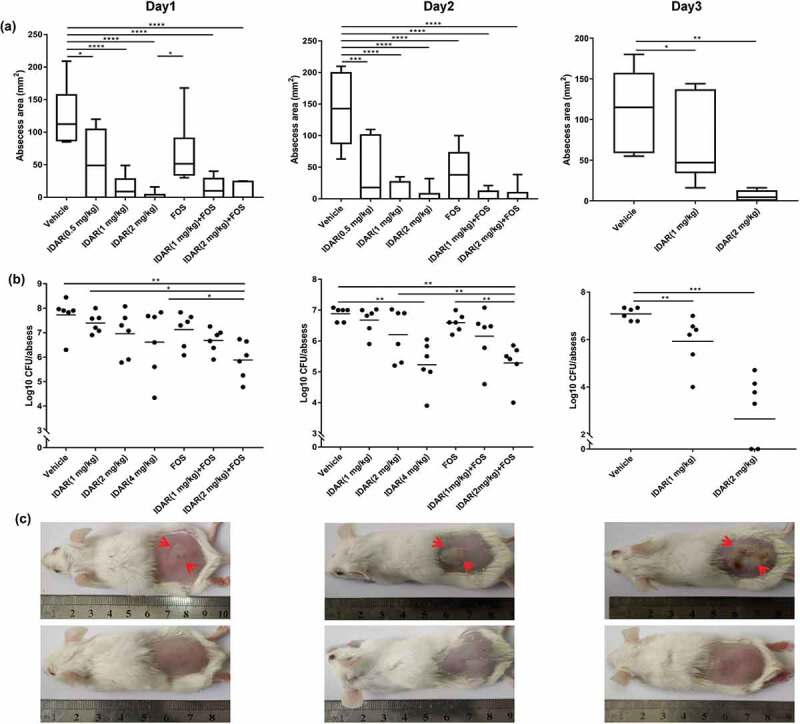
IDAR synergy with FOS eradicates MRSA in a subcutaneous abscess mouse model. Size (a) and bacterial load (b) of abscesses after treatment with IDAR (1–4 mg/kg, s.c.) in the presence/absence of FOS (20 mg/kg, s.c.) for 3 d. (c) Representative images of subcutaneous abscesses before and after IDAR treatment.

We constructed a subcutaneous abscess mouse model by subcutaneously injecting a high density (approximately 10^8^ CFU/mouse) of MRSA. Then, the antimicrobial efficacy of IDAR alone or in combination with FOS was assessed by subcutaneously injecting one dose. We found that a single dose of IDAR (ranging from 0.5 to 2 mg/kg) could significantly inhibit abscess formation within 3 d after treatment in a dose-dependent manner. FOS also showed moderate MRSA abscess inhibitory effects. When FOS was combined with IDAR, the abscesses were significantly diminished. However, we could not judge the synergistic effects of the two drugs together because of the obvious efficacy of IDAR used alone (Figure 6(a)). A single dose of IDAR or FOS could not eradicate the bacteria in the abscess, as measured by bacterial cell counts on the first day after treatment, but there were significant synergistic antimicrobial effects between the two drugs, as demonstrated by the decreased bacterial load of 1.84 ± 0.77 CFU/abscess. IDAR at 4 mg/kg began to show significant antimicrobial effects on d 2, and when combined with FOS, only 2 mg/kg of IDAR was needed to eradicate the bacterial load in the abscess. Furthermore, the bacterial eradicating effects were amplified on d 3, at which time, only a single dose of IDAR at 1 mg/kg showed significant eradication of the bacterial load in the abscess, and 2 mg/kg of IDAR could reduce the number of bacterial cells by 4.43** **± 2.11 CFU/abscess (Figure 6(b)). In accordance with the description above, images showed that 2 mg/kg IDAR treatment had strong abscess inhibitory effects (Figure 6(c)).

**Figure 7.**
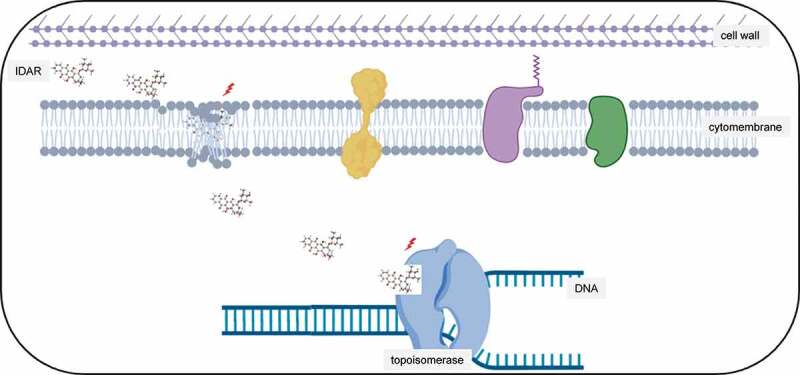
Antimicrobial model of the mechanism of IDAR. Red lighting indicates the target of IDAR. IDAR penetrates the cell wall into the cell membrane, disrupts the normal structure of the phospholipid bilayer, and in addition, some IDAR penetrates the cytoplasm and interacts with the topoisomerase IIA subunits GyrA and GyrB, inhibiting DNA replication and cell growth and showing bactericidal effects.

### Postantibiotic effects of IDAR against *S. aureus*

PAE and PAE-SME assays were performed to assess the postantibiotic effects of IDAR. The average PAE values for IDAR treatment of ATCC 43300 and ATCC 29213 were 6 h, while the PAE values were 3 and 4 h for VAN treatment, respectively. This indicates that IDAR had better postantibiotic effects than VAN against *S. aureus* compared to VAN. Similarly, sub-MIC levels (1/2, 1/4, and 1/8 × MIC) of IDAR also exhibited better postantibiotic effects compared to VAN, with resulted in a longer period of time in which live bacteria could be recovered (Table 4).

**Table 4.** PAEs of inhibitory and subinhibitory concentrations of the antimicrobial compounds tested in this study.

Strain	MIC (μg/ml)	PAE (h)		PAE-SME (VAN, h)			PAE-SME (IDAR, h)		
		VAN	IDAR	1/2× MIC	1/4× MIC	1/8× MIC	1/2× MIC	1/4× MIC	1/8× MIC
ATCC43300	4	3	6	>12	6	6	>12	8	7
ATCC29213	4	4	6	10	10	8	>12	>12	8

## Discussion

In this article, the antitumor drug IDAR showed strong antimicrobial effects against both MRSA and *S. epidermidis* by targeting the cell membrane and the topoisomerase IIA subunits GyrA and GyrB (Figure 7). To the best of our knowledge, there have been no studies reporting the mechanisms underlying the antibacterial effects of IDAR. Bacterial infection, particularly in patients with hematologic malignancies, is the most common complication of chemotherapy-induced neutropenia [36]. Because IDAR has both antimicrobial and anticancer effects, it may be suited for the treatment of hematologic patients with *Staphylococcus* sp. infections.

IDAR shows significant antimicrobial effects against MRSA and MRSE clinical isolates with different antibiotic resistance phenotypes. And this indicates that the target of IDAR is different from traditional antibiotics. Similarly, Hou et al. [37] found that IDAR had antiviral effects at the micromolar level against enterovirus species, probably through its ability to block the synthesis of viral proteins and RNA, thereby suppressing virtual ribosomal entry site-mediated translation. Using aerobic BacT/Alert standard as the culture media along with FAN bottles, Kinnunen et al. [38] also found that IDAR could inhibit some gram-positive cocci, as well as *Candida* sp. growth.

The bacterial cell membrane is an important target for antimicrobial drug development because it can be disrupted independently of growth. The gram-positive bacterial envelope possesses a thick peptidoglycan layer that is enriched in negatively charged teichoic acids and a single lipid membrane [32]. Many studies have proven the antimicrobial effects of repurposed drugs that target the bacterial lipid membrane, such as the antitumor drug CD437 and CD1530 [39] and the anthelminthic drug bithionol [23]. Similarly, in our present study, we observed disruption of the cell membrane by an all-atom MD simulation, TEM observation, and SYTOX Green permeability assay, indicating that the cell membrane is the potential target of IDAR. The development of antimicrobials that targeting the cell membrane has many advantages such as antipersister activity, fast killing, and a low occurrence of the development of resistance [40]. However, for gram-negative bacteria, the outer membrane contains negatively charged lipopolysaccharides and an inner cytoplasmic membrane surrounded by a peptidoglycan layer; therefore, it may be hard for IDAR to transmit into the inner cell membranes [32]. In accordance with our results, PMBN could enhance bacterial cell membrane permeability and therefore increase the susceptibility of gram-negative strains. Although IDAR had a good docking score with the GyrA/GyrB proteins of the type IIA topo by bioinformatic analysis, IDAR only showed modest affinity and inhibitory effects with the DNA gyrase. Thus, the main mechanism underlying its antimicrobial activity of IDAR is cell membrane disruption rather than DNA gyrase inhibition.

The antineoplastic mechanism of IDAR is mainly attributed to its DNA intercalating ability and its inhibition of topo II impairs cellular DNA replication [37]. IDAR may cause cytotoxicity to normal cells when treating infectious diseases. Thus, the topical use of IDAR may be a better choice compared to systemic use. In the treatment of leukemia, IDAR is typically administered in a dose of 10 ~ 15 mg/m^2^ for a couple of days, depending on the regimen where it is being used [41,42]. Mice have an average body weight/surface area ratio of ~3 kg/m^2^. Therefore, the dose of 1 ~ 2 mg/kg used in the current experiment is related to 3 ~ 6 mg/m^2^ and is below the lowest range used for human chemotherapy [41]. Different from the administration in leukemia patients, short term or even a single dose of IDAR therapy could significantly reduce the bacterial load in an abscess or infected wound, which may result in decreased cytotoxicity in the human body. Furthermore, because we observed a synergistic antimicrobial effect between IDAR and FOS, a drug combination may also be a good choice to reduce the necessary dose of IDAR, thereby lowering its cytotoxicity. In addition, because IDAR mainly targets the bacterial cell membrane, targeting the bacteria via multiple mechanisms leads to a low occurrence of resistance. The most common side effect of IDAR is cardiotoxicity [43]. The production of oxygen radicals induced by IDAR has often been considered as a molecular base of heart failures. However, some studies argue that the first step in cardiac myocyte damage from anthracyclines is not the production of oxygen radicals but by targeting the Top2β [14,44]. AMI, which is the only FDA-approved cell protector, was found to decrease the side effects of IDAR in normal cells, but not cancer cells, by acting as a free radical scavenger and accelerating the DNA repair process [43,45]. In our study, AMI significantly reduced the cytotoxicity of IDAR without influencing bacterial growth. The combination of AMI and IDAR also showed no liver, kidney, or cardiotoxicity *in vivo*. Besides AMI, there are also a great amount of drugs that could be used to prevent IDAR-evoked cytotoxicity. For example, dexrazoxane is approved by FDA to prevent cardiotoxicity and genomic damage caused by IDAR [41]; Vitamin C could be used as protective agents against DNA damage in normal cells in the presence of IDAR [43]; Theanine and 1-methyl-3-propyl-7-butylxanthine could increase the antitumor activity of IDAR and ameliorates its toxicities [46]. Grape seed proanthocyanidin extract can ameliorate the toxic effects of IDAR by upregulating the expression of Bcl-2 [47]; Other agents such as Vitamin E, Coenzyme Q10, carnitine, probucol, carvedilol, et al. also have the ability to reduce the cardiotoxicity caused by IDAR [48]. Therefore, cell protectors make it possible for IDAR in the treatment of infectious diseases in clinical settings. Besides, another side effect of anthracyclines is therapy-related myeloid neoplasms (t-MNs) with chromosomal band t(4; 11)(q21; q23) [49]. But the occurrence of t-MNs is relatively infrequent and needs long-term anthracycline treatment, and the 6-y cumulative incidence of t-MN is only 2.2% [50]. In our present study, the treatment period of IDAR as antimicrobials is within 3 d, which has a low possibility to induce t-MNs.

IDAR could significantly inhibit the formation of subcutaneous abscesses caused by a high load of MRSA within 3 d. There are few research studies on infections that are associated with high bacterial loads (more than 10^7^ CFU/ml), especially abscess-related infections, which are difficult to treat and can lead to increased resistance to conventional antibiotics [51]. In our present study, a single dose of IDAR by subcutaneous injection could significantly inhibit the formation of abscesses on d 1 after treatment. However, IDAR did not begin to eradicate the bacterial load in abscesses until d 2. The abscess inhibitory effects of IDAR are partly due to the anti-inflammatory effects of IDAR, as well as the inhibition of bacteria growth and the release of virulence factors. IDAR may also cooperate with immune cells, such as neutrophils, which can easily kill the bacterial cells in an abscess.

By structure–activity relationship analysis, we found that other anthracycline drugs also have potential inhibitory effects against MRSA (Table 5). As reported everywhere, structurally similar molecules often have similar drug efficacies, such as CD437 and CD1530 [39], bithionol and its analogs [23], and G0775 and its analogs [52]. This indicates that the parent structure of anthracene ketone is the main effector in its antimicrobial efficacy, and the exploration of anthracene ketone-based analogs with better antimicrobial effects and lower cytotoxicity is very attracting. Of course, to develop as an antibiotic, the structure of IDAR also should be optimized to ameliorate side effects and keep its antimicrobial effects. For example, analog synthesis, molecular hybridization [53], local delivery systems (such as siderophores, antimicrobial peptides, antibodies, and nanoparticles) [54,55], and drug combination, et al.

**Table 5.** MIC and MBC of anthracycline drugs against *S. aureus.*

		ATCC43300		ATCC29213	
Drugs	Structure	MIC (μg/mL)	MBC (μg/mL)	MIC (μg/mL)	MBC (μg/mL)
IDAR		4	8	4	16
Pirarubicin		4	>128	8	128
Doxorubicin		8	>128	16	>128
Epirubicin		4	>128	8	>128
Daunorubicin		4	>128	8	128

In conclusion, IDAR shows potential anti-MRSA, antipersister, and antibiofilm activity through its targeting of the cell membrane. IDAR could be used for the treatment of MRSA-related wound infections and subcutaneous abscesses. Finally, it is important to realize the long-term potential of further chemical optimization of IDAR in the development of nontoxic antimicrobials.

## Importance

With the emergence of linezolid and vancomycin-resistant MRSA strains sporadically reported worldwide, the development of new antimicrobial agents against multi-drug resistant strains is extremely important. Repurposing approved drugs is a promising alternative strategy, which has the ability to reducing cost and time and accelerating the process of antibiotic research and development. In the present study, we repurposed the anti-tumor agent idarubicin as an antimicrobial, which shows excellent bactericidal and anti-persister effects against MRSA strains. Hence, with the development of improved idarubicin analogs, anthracyclines have the potential to become a new class of antibiotics.

## Supplementary Material

Supplemental Material
