# Supplementary figures and images for: Insights into idarubicin antimicrobial activity against methicillin-resistant Staphylococcus aureus
Source: Virulence. 2020 May 29;11(1):636–51. doi: 10.1080/21505594.2020.1770493 (PMC7549941; doi:10.1080/21505594.2020.1770493)

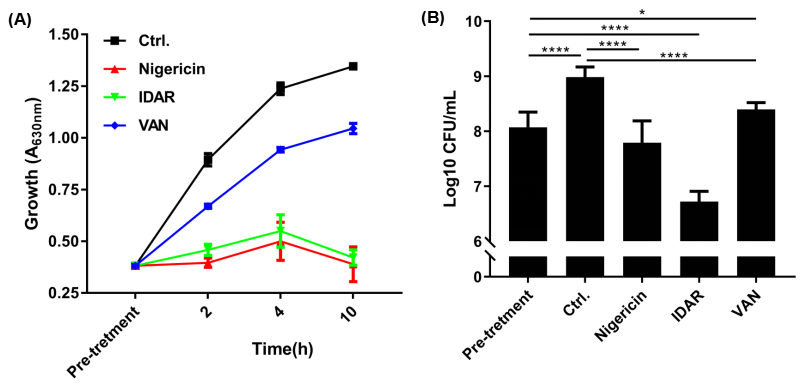

Supplement: Supplemental Material [file KVIR_A_1770493_SM9471.zip › Fig. S1.tif]

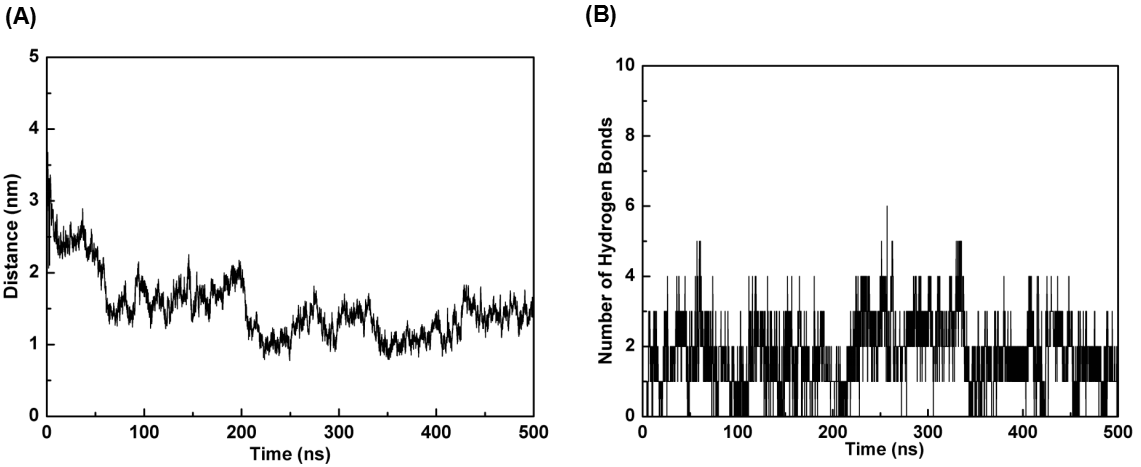

Supplement: Supplemental Material [file KVIR_A_1770493_SM9471.zip › Fig. S2.tif]

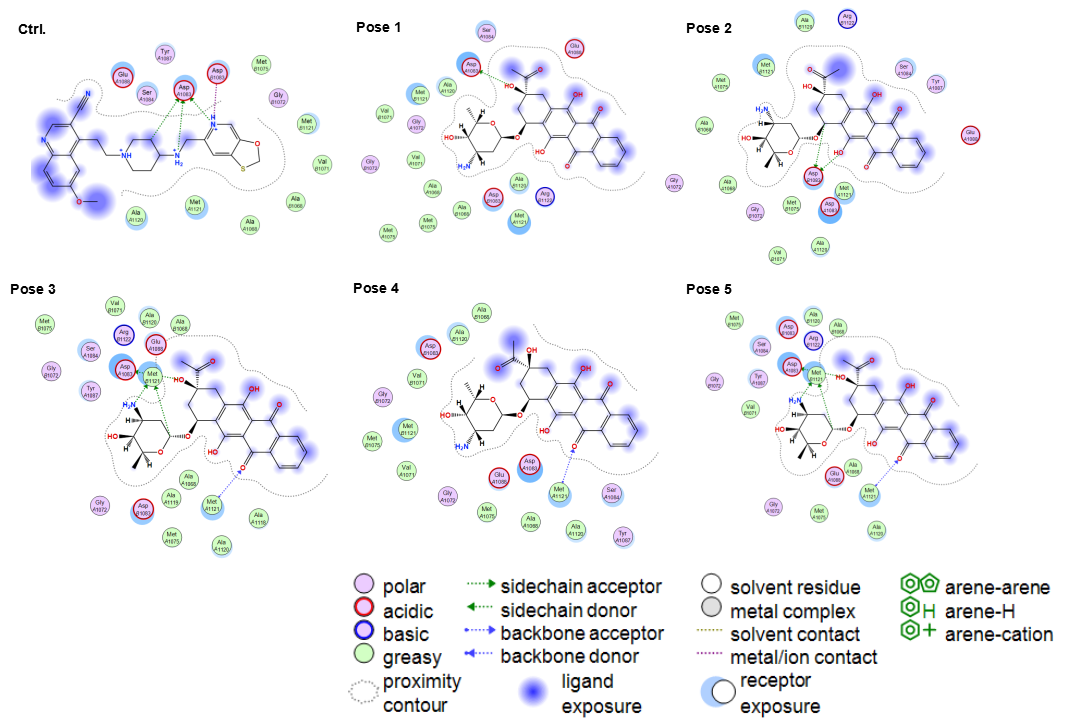

Supplement: Supplemental Material [file KVIR_A_1770493_SM9471.zip › Fig. S3.tif]

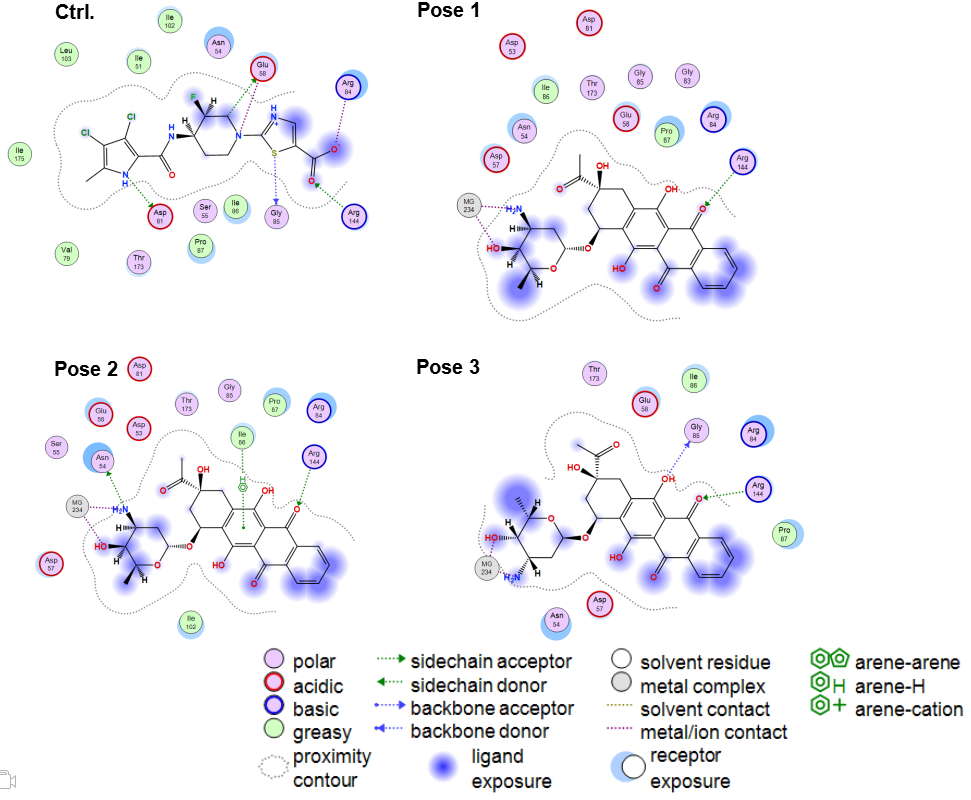

Supplement: Supplemental Material [file KVIR_A_1770493_SM9471.zip › Fig. S4.tif]

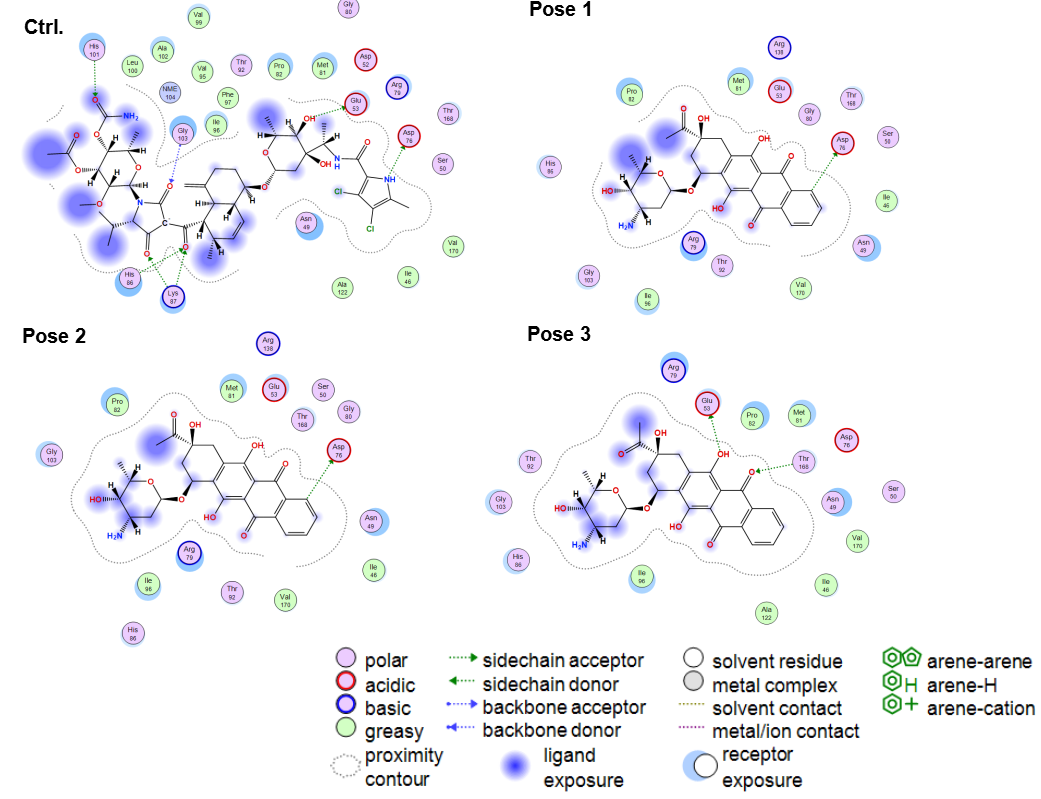

Supplement: Supplemental Material [file KVIR_A_1770493_SM9471.zip › Fig. S5.tif]
