# Supplementary material for: Insights into idarubicin antimicrobial activity against methicillin-resistant Staphylococcus aureus
Source: Virulence. 2020 May 29;11(1):636–51. doi: 10.1080/21505594.2020.1770493 (PMC7549941; doi:10.1080/21505594.2020.1770493)
Supplement: Supplemental Material [file KVIR_A_1770493_SM9471.zip › 20200202 Supplemartory data.doc]

**Supplementary data**

**Supplementary methods**

***1. Bacterial strains used in this study***

The type strains including *S. aureus* ATCC 25923 and ATCC 29213, *Enterococcus faecalis* ATCC 29212, *Acinetobacter banumannii* ATCC 1195, *Escherichia coli* ATCC 25922, *Klebsiella pneumonias* ATCC 700603 and *Candida albicans* ATCC 14053 were kindly provided by Juncai Luo (Tiandiren Biotech, Changsha, China). *S. aureus* ATCC 43300 and Newman were kindly provided by Min Li (Renji Hospital, School of Medicine, Shanghai Jiaotong University). *Staphylococcus epidermidis* RP62A and ATCC 12228 were kindly provided by Qu Di (Laboratory of Medical Molecular Virology, Shanghai Medical College, Fudan University, Shanghai, China). *Pseudomonas aeruginosa* PAO1 was kindly provided by Qiao Minqiang (College of Life Sciences of Nankai University, Tianjin, P.R. China). Clinical strains of *S. aureus* were isolated form the Third Xiangya Hospital of Central South University (She et al., 019). *S. aureus* and *S. epidermidis* strains were grown in tryptic soy broth (TSB) (Solarbio, Shanghai, China). *E. faecalis* strains were grown in brain-heart infusion (BHI) broth (Solarbio, Shanghai, China). *C. albicans* strains were grown in RPMI 1640 (Gibco, ThermoFisher, USA) and gram negative species were grown in Luria Bertani (LB) broth (Solarbio, Shanghai, China). All bacteria were grown at 37 ℃, 180-200 rpm.

***2.Molecular docking***

Briefly, molecular docking was performed in MOE v2018.0101 [Molecular Operating Environment (MOE), 2018.01; Chemical Computing Group Inc., 1010 Sherbooke St. West, Suite #910, Montreal, QC, Canada, H3A 2R7. 2018]. The 2D structures of IDAR were drawn in ChemBioDraw 2014 and converted to 3D structures in MOE through energy minimization. The structures of the topoisomerase IV and topoisomerase IIA subunit GyrA and GyrB proteins were downloaded from the RCSB Protein Data Bank [the PDB IDs were 4URL (topoisomerase IV), 2XCS (GyrA subunit) and 3TTZ (GyrB subunit)]. Prior to docking, the force field of AMBER10: EHT and the implicit solvation model of the Reaction Field (R-field) were selected. The original ligand binding sites in the proteins were selected as the molecule binding pocket. The docking workflow followed the “induced fit” protocol, where the side chains of the receptor pocket were allowed to move according to ligand conformations, with a constraint on their positions. The weight used for tethering side chain atoms to their original positions was 10. For each ligand, all docked poses were ranked by London dG scoring first, then a force field refinement was carried out on the top 30 poses, followed by a rescoring of GBVI/WSA dG. The conformations with the lowest free energies of binding were selected as the best binding modes. PyMOL (www.pymol.org) was used to generate molecular graphics.

***3. Process of Surface Plasmon Resonance (SPR)***

pH scouting was performed with 10 mM NaAC at a series of pH values, and 4.5 was chosen as the optimal pH for the following experiments. To activate the chip surface, an EDC:NHS solution (1:1, NHS 0.05 M + EDC 0.2 M) was injected into the sensor chip for 7 min. For protein immobilization, the protein solution was injected into channel 1 for 10 min and HBS-P buffer with 2% DMSO was injected (running buffer) into channel 2 (reference channel). Finally, ethanolamine-HCl (1 M, pH 8.5) was injected to block the remaining activated surface groups. All of the injection processes were performed with a flow rate of 30 μl/min. IDAR was injected at various concentrations (0.781-12.5 μM) and the flow cell without any protein was used as a reference. The affinity was analyzed by Biacore S200 Evaluation Software (Sharifi et al., 2017).

***4. Transmission Electron Microscope (TEM)***

Samples were fixed with the same volume of 2 × fixative (5% glutaraldehyde, 2.5% paraformaldehyde and 0.06% picric acid in 0.2 M sodium cacodylate buffer, pH 7.4). Fixed cells were washed in 0.1 M cacodylate buffer and postfixed with 1% osmium tetroxide/1.5% potassium ferrocyanide for 1 h. The fixed cells were washed in water and maleate buffer and then incubated in maleate buffer containing 1% uranyl acetate for 1 h. The bacterial cells were then washed twice in water and subsequently dehydrated in an alcohol gradient series. The cells were then incubated in propyleneoxide for 1 h, infiltrated overnight in a 1:1 mixture of propyleneoxide and Spurr’s low viscosity resin and polymerized at 60 ℃ for 48 h. Ultrathin sections (approximately 60 nm) were cut on a Reichert Ultracut-S microtome, picked up onto copper grids, and stained with lead citrate. Micrographs of the cells were taken using a transmission electron microscope (HITACHI HT-7700, Japan) (Kim et al., 2019).

**5.*Animals***

***Acute skin infection model***

Specific pathogen-free C57 female mice (Hunan, China), aged 9 weeks and weighing 18-20 g were used. Mice were anesthetized by intraperitoneal (i.p.) injection of sodium pentobarbital (50 mg/kg.) and their backs were shaved by an electric razor. Mice were kept under anesthesia and wounds of an area of approximately 2 cm^2^ were made using a sterile needle. Then, 5 μl of an *S. aureus* suspension was loaded onto the wounded skin. Two hours after infection, ointment containing 0.8% IDAR was applied to the skin and covered with a small piece of parafilm. The vehicle (0.8% DMSO) was applied to the skin as a control. After treatment for 8 h, the skin was excised and homogenized in 1 ml of saline. The number of viable cells were counted (de Breij et al., 2018).

***Murine subcutaneous abscess model***

Specific pathogen-free CD-1 female mice (Hunan, China), aged 7 weeks and weighing 22-28 g were used. Mice were anesthetized with sodium pentobarbital (50 mg/kg, i.p.) and their backs were shaved by an electric razor. Prior to injection, bacterial cells were washed twice with saline and resuspended in saline. A 50 μl suspension containing 1 × 10^8^ CFU cells was subcutaneously injected into the dorsum. After infection for 1 h, 50 μl amifostine (AMI) (25 mg/kg) was injected subcutaneously to release the cytotoxicity of IDAR. Then, 30 min after treatment, the mice were treated subcutaneously with 100 μl of IDAR (1-4 mg/kg) in the presence/absence of FOS (20 mg/kg). The progression of the infection was monitored daily and abscesses were excised and homogenized in saline. Viable cell counts were performed as described above (Pletzer et al., 2018).

***6.Resistance induction assay***

***Drug resistance screening by sub-MIC induction***

MRSA ATCC 43300 was cultured overnight and diluted in TSB broth, then cultured to log-phase. The suspensions were diluted with MH broth to a final concentration of 2 × 10^5^ CFU/ml. Fifty microliters of the suspension was added to an equal volume of 2-fold diluted IDAR in microplates with 2 parallel tests (P1 and P2). CIP was included as a positive control. After incubation at 37 ℃ for 24 h, the MIC was read as described by CLSI 2012. The bacterial suspension at which cells grew in the presence of the highest concentration of IDAR was diluted 1:1000 with MH broth. This bacterial suspension was used again to perform the assay for a total of 16 days (Kim et al., 2018) .

***Frequency of resistance***

A single colony was cultured overnight in TSB broth and then the cells were collected by centrifugation at 3000 g for 15 min. Then, approximately 3 × 10^9^ CFUs were spread on cation adjusted MH agarose plates containing IDAR at a final concentration of 1 - 8 times the MIC. After incubation at 37 ℃ for 48 h, viable cells were counted. The frequency of resistance was defined as the number of colonies that formed after 48 h, divided by the initial viable cell count (Smith et al., 2018).

***7.Postantibiotic effect and postantibiotic sub-MIC effect***

For postantibiotic effect (PAE) testing, tubes containing 5 ml of MH broth with 10 times the MICs of the tested antimicrobials were inoculated with approximately 5 × 10^6^ CFU/ml. Inoculation with DMSO was included as a growth control. The test tubes were then placed in an incubator at 37 ℃ at 180 rpm for 1 h. After incubation, cultures were diluted 1:1000 in MH broth to remove the effect of the antimicrobials. Viable cell counts were determined by the plate dilution method every 2 h until the turbidity reached approximately equivalent to 1 McFarland standard. The PAE was defined as T - C (T indicates the time required for the viable count of the test group to increase above the count obtained immediately after dilution by 1×log10; C indicates the related time for the control group).

For postantibiotic sub-MIC effect (PAE-SME), after a 1:1000 dilution as described above, antimicrobials were added into the tubes to final concentrations of 0.125 ×, 0.25 ×, and 0.5 × MIC. Tubes with DMSO were included as a control. Viability counts were determined as described above. The PAE-SME was defined as T_pa_ - C (T_pa_ indicates the time required for the cultures exposed to antimicrobials followed by sub-MIC antimicrobials to increase above the count immediately after dilution by 1×log10; C indicates the related time for the control group). The PAE and PAE-SME were repeated two times on independent days (Pankuch et al., 2009).

***8.Red blood cells hemolysis***

Fresh horse RBCs were rinsed with PBS three times and diluted to a final concentration of 4%. After incubation at 37 ℃ for 10 min with shaking, 200 μl of the suspensions were added to a microplate, and then a series of IDAR concentrations were added to each well. RBC suspensions treated with 2% DMSO and Triton X-100 (1%) were included as 0% and 100% hemolysis controls, respectively. The suspensions were then incubated at 37 ℃ for 1 h. After centrifugation for 10 min at 800 × g, hemolysis activity was measured as the absorbance of the supernatant at 540 nm (A540).

***9.Cytotoxicity assay by CCK-8***

HcoEpiC, BEL-7404 and human skin fibroblasts (HSF) cell lines were cultured in DMEM or RPMI1640 with 10% Gibco FBS, respectively. One hundred microliters of log-phase cells were added to a microplate at a final concentration of 3 × 10^3^ (for HcoEpiC and BEL-7404) or 7 × 10^3^ (for HSF) cells/well. Following overnight incubation and adherence at a 37 ℃ with 5% CO_2_ in a humidity controlled incubator, cells were treated with IDAR at concentrations ranging from 1 to 128 μg/ml, with cells treated with 0.3% DMSO included as a control. After incubation under normal tissue culture conditions for 8 h, a CCK-8 kit was used to determine the cell activity according to the manufacturer’s instructions. The IC50 was calculated using GraphPad 8.0 software (Smith et al., 2018).

***10. Statistical analysis***

All data were analyzed using GraphPad Prism 8.0 software. To examine the significant differences between groups, the data were compared using a Student *t* test, whereas data comparisons across more than two groups were performed using a one-way analysis of variance followed by Tukey tests or Dunn’s multiple comparison test. *p* < 0.05 was considered statistically significant. *: p<0.05; **: p<0.01; ***: p<0.001; ****: p<0.0001.

**Supplementary references**

de Breij A, Riool M, Cordfunke RA, et al. The antimicrobial peptide SAAP-148 combats drug-resistant bacteria and biofilms. *Sci Transl Med* 2018; **10**: eaan4044.

Sharifi M, Dolatabadi JE, Fathi F, *et al*. Kinetic and thermodynamic study of bovine serum albumin interaction with rifampicin using surface plasmon resonance and molecular docking methods. *J Biomed Opt* 2017; **22**: 37002.

She P, Zhou L, Li S, *et al*. Synergistic microbicidal effect of auranofin and antibiotics against planktonic and biofilm-encased *S. aureus* and *E. faecalis*. *Front Microbiol* 2019; **10**: 2453.

Kim W, Hendricks GL, Tori K, *et al*. Strategies against methicillin-resistant Staphylococcus aureus persisters. *Future Med Chem* 2018; **10**: 779-94.

Kim W, Zou G, Hari TPA, *et al*. A selective membrane-targeting repurposed antibiotic with activity against persistent methicillin-resistant *Staphylococcus aureus*. *Proc Natl Acad Sci U S A* 2019; **116**: 16529-34.

Pankuch GA, Appelbaum PC. Postantibiotic effect of ceftaroline against gram-positive organisms. *Antimicrob Agents Chemother* 2009; **53**: 4537-9.

Pletzer D, Mansour SC, Hancock REW. Synergy between conventional antibiotics and anti-biofilm peptides in a murine, sub-cutaneous abscess model caused by recalcitrant ESKAPE pathogens. *PLoS Pathog* 2018; **14**: e1007084.

Smith PA, Koehler MFT, Girgis HS, *et al*. Optimized arylomycins are a new class of Gram-negative antibiotics. *Nature* 2018; **561**: 189-94.

**Supplementary Figures**

**Figure S1**. IDAR inhibits MRSA growth even at a high density. (A) Time-growth curve of MRSA at the start of inoculation (OD630 of approximately 0.38, or approximately 10^8^ CFU/ml) in the presence of VAN (10 × MIC, negative control), NI (10 × MIC, positive control), and IDAR (10 × MIC). (B) Viable cell counts at 10 h timepoint.

**Figure S2.** The change of the centroid distance (A) and the number of hydrogen bonds (B) between IDAR and phospholipid membrane with simulation time within 500 ns.

**Figure S3.** Interaction model of IDAR and 2XCS in different poses by molecular docking. Ctrl: intrinsic legend of 2XCS.

**Figure S4.** Interaction model of IDAR and 3TTZ in different poses by molecular docking. Ctrl: intrinsic legend of 2XCS.

**Figure S5.** Interaction model of IDAR and 4URL in different poses by molecular docking. Ctrl: intrinsic legend of 2XCS.

**Supplementary Video 1.** Molecular dynamics of IDAR interacting with mixed lipid bilayers at a DOPC:DOPG ratio of 7:3. In the videos, IDAR is depicted as large spheres, and phospholipids are represented as chains.
